# Effectiveness of a Video-Feedback and Questioning Programme to Develop Cognitive Expertise in Sport

**DOI:** 10.1371/journal.pone.0082270

**Published:** 2013-12-10

**Authors:** Luis García-González, M. Perla Moreno, Alberto Moreno, Alexander Gil, Fernando del Villar

**Affiliations:** 1 Faculty of Health and Sport Sciences, University of Zaragoza, Huesca, Spain; 2 Faculty of Sport Sciences, University of Extremadura, Cáceres, Spain; Universidad de Granada, Spain

## Abstract

The importance within sport expertise of cognitive factors has been emphasised in many research studies. Adaptations that take place in athletes’ long-term memories are going to condition their decision-making and performance, and training programmes must be developed that improve these adaptations. In our study, we provide a tactical-cognitive training programme based on video-feedback and questioning in order to improve tactical knowledge in tennis players and verify its effect when transferred to athletes’ decision-making. 11 intermediate tennis players participated in this study (12.9±0.7 years old), distributed into two groups (experimental, *n* = 5; control, *n* = 6). Tactical knowledge was measured by problem representation and strategy planning with a verbal protocol. Decision-making was measured by a systematic observation instrument. Results confirm the effectiveness of a combination of video-feedback and questioning on cognitive expertise, developing adaptations in long-term memory that produce an improvement in the quality of tactical knowledge (content, sophistication and structure). This, in turn, is transferred to the athletes’ decision-making capacity, leading to a higher percentage of successful decisions made during game play. Finally, we emphasise the need to develop effective programmes to develop cognitive expertise and improve athletes' performance, and include it in athletes’ formative stages.

## Introduction

We can find many research studies within the field of expert performance that emphasise the influence of cognitive elements on sport expertise (see [1]–[3] for a review). From the cognitive psychology approach, expertise level in a certain sport depends on inner mental representations and on the cognitive processes that lie between stimulus interpretation and action selection [4]. The athlete’s limited knowledge will determine cognitive processes such as decision-making in sport [5].

There is a strong interrelationship between the different elements that comprise cognitive expertise. Knowledge structures stored in our memory are a constraint to decision-making [6]. The greater and more varied this knowledge is, the better the anticipation and decision-making will be in athletes [7]–[8]. Knowledge has an influence on other cognitive processes, such as directing attention, visual behaviour, anticipation as well as response selection, decision-making and execution or performance. Likewise, the knowledge accessed and the use of strategies and tactics will depend on the context determined by the environment, the athlete and the task [9]. Furthermore, the development of procedural knowledge will permit the development of processes that determine when intuitive decisions are necessary and when deliberate decisions are more adequate [10], as both deliberate decisions and intuitive decisions exist within a continuum of consciousness [11].

Related to deliberate decision-making and with reference to knowledge and internal processes, as the sport expertise level increases, we can find greater development of specific adaptations and structures, stored in the long-term memory (LTM). Thus, when a decision has to be made during a real match situation, an expert tennis player will make a better decision, because of two specialised adaptations of LTM: i) action plan profiles, which are rules stored in the LTM, and are used to make the current environmental conditions coincide with the motor actions, including cognitive skills, to supervise current conditions; ii) and current event profiles, which are tactical scripts that guide the construction and constant change of important concepts, which are going to be taken into account in subsequent actions (e.g. updating information about the opponent’s strong points during a game). Current event profiles are constructed on the basis of previous competitions, previous experiences, or with cognitive skills to collect data as the competition advances (for further review of this model, [6], [9], [12]–[13]). Both profiles are predicted to allow players easy access to and retrieval of important information to make decisions during competition and to compensate or make adjustments during time-constrained moments [6].

The schemes related to decision-making (e.g., action plan profiles) will allow players to quickly and efficiently plan and programme responses [14]. The use of the Long-Term Working Memory [15] can also be important because it is an additional mechanism to access relevant tactical information by means of adaptations in the LTM. These adaptations enable information to be coded and recovered [9]. Specialised profiles in the LTM (e.g., action plan profiles and current event profiles) will make access to relevant tactical information easier. This will also help athletes take better and more successful decisions in real game play situations [6], [12]–[13].

These recovery structures will enable the information to be classified and stored, in order to carry out a superior representation about game scenarios, facilitating recognition and anticipation vis-à-vis game events [16]. A dynamic coding of the environment will integrate recovery structures in “real time” where experts can anticipate future events, predict results and develop proactive and reactive behaviours by developing action plans [17].

Research within the expert-novice approach may help understand and comprehend sport expertise and expert performance [18]. Research carried out in tennis, within the expert-novice paradigm, presents remarkable differences in content, sophistication and conceptual structure of the expert players’ knowledge. Knowledge is greater, more varied and sophisticated. Furthermore, these developed concepts are related to each other and favour greater knowledge structuring [6], [13], [19]–[22]. In addition, better tennis playing is caused by better temporal processing [23]. Consequently, it is possible to find a considerable relationship between expertise level, knowledge, tactical decision-making skills and performance in sport [24]–[25]. All these cognitive characteristics enable expert tennis players to make more suitable decisions based on superior mental representations [6], it being necessary to develop the different cognitive processes related to performance. The superior expertise level of expert athletes is made evident through their mental representations and their cognitive processes (e.g. decision-making). These elements indicate performance in sport [4].

In open sports, the knowledge basis changes when the expertise level increases, developing different modifications in content, sophistication and knowledge structure: a) action plans without a hierarchically defined structure are replaced by conditions and actions that act as decision rules; b) weak or unsuitable conditions and actions become tactical and associated conditions and actions; c) approaches to global sport situations, with minimum processing of relevant task elements, are replaced by more tactical approaches, with relevant information (including past events and current facts); d) processing environmental events or superficial characteristics is replaced by the in-depth processing of information [13], [22], [26].

One of the difficulties found here is the lack of research about how to produce these adaptations, apart from those developed through hours of practice. The last step in the expert performance approach establishes the need to examine the acquisition of identified expertise characteristics using training interventions [27]. That is why we wonder: Which activities may favour the development of LTM adaptations to improve cognitive skills (e.g., tactical knowledge and decision-making)?

There are some activities directly related to performance improvement, such as video training or individual instructions [28], [29]. Specific research on the development of cognitive factors is much more limited, although we find some approaches to develop factors related to cognitive expertise (see [30] for a review).

To develop tactical-decisional skills and cognitive skills in sport, the main goals are to promote and construct tactical experiences. Vickers [31] develops a Decisional Training Model (DTM), and points to the use of tools such as video-feedback and questioning [30]–[32]. The use of video-feedback is also recommended to analyse tactical behaviour (own and opponent’s) during competition, to develop situation profiles, to develop tactical knowledge in athletes, or to improve the recognition of contextual factors [10], [33]. Consequently, video-feedback seems to be a useful tool to develop cognitive expertise, although the help of an expert in its use and interpretation is recommended to find benefits. Showing videos to athletes about themselves has limited results, because recorded events happen too fast and there is a lot of available information [31]. On the other hand, when the athlete's attention is directed by a supervisor towards specific signs through questioning, results are significantly positive [31], [34]. However, there is a little research in the combined use of both decisional tools (i.e., video-feedback and questioning). This combination of tools for training could be defined as an explicit learning strategy. This explicit learning, defined as an intentional acquisition which produces verbalisable knowledge, may be focused on which signals could be used and how to use them to get results or to make decisions in sport [35]–[36]. Furthermore, decisional learning, which begins through deliberate, intentional and explicit procedures, may become automated and intuitive with time [37].

The use of video-feedback and questioning programmes is recommended for the cognitive development of athletes, especially at lower performance levels, where this could be more effective [38]–[39]. That is why our interest lies in examining and confirming the effect of a combined video-feedback and questioning programme on the cognitive expertise and decision-making of tennis players. The basic hypothesis suggests that the application of a tactical-cognitive training programme based on video-feedback and questioning will have a significant positive effect on the tactical knowledge of tennis players, on its content, sophistication and conceptual structure. In addition, an improvement of athletes’ cognitive expertise will produce an increase in decision-making skills with a higher percentage of successful decisions made during real game play.

## Methods

### Participants

11 male players took part in this research, and they were divided into two groups: control (*n* = 6) and experimental (*n* = 5). One player, who was initially included in the experimental group, comprised of six players, was removed because of an injury produced during this study. All athletes belonged to the Under-14 category. To ensure that all players would be exposed to the same formative process, all players belonged to the same club, to the same training group, they had the same trainer and all of them trained 4 hours per week. All of them had the same classification according to the ITF (International Tennis Federation), by means of the International Tennis Number (ITN). They were also included in level 5-Intermediate.

The subdivision into two groups (experimental and control) was done according to the evaluation of the decision-making level, by means of the observation protocol of Nielsen & McPherson [24]. This evaluation was done in 4 complete matches, as a pre-test, and before the start of the intervention (see Figure S1). Another criterion to form the groups was tactical knowledge obtained in the pre-test. Tests for variance homogeneity were performed on all the aforementioned variables by means of the Levene test. Levene’s statistic was not significant in problem representation variables (*p* >. 05 in all variables), planning strategies variables (*p* >.05 in all variables) or decision-making (*p* >.05). This showed the equivalence of groups before starting the intervention.

Characteristics such as age, years of practice and years of competition in tennis of each group are included in Table 1.

**Table 1:** Characteristics of participants in every group.

Variables	Control Group (n = 6)		Experimental Group (n = 5)	
	M	SD	M	SD
Age	12.83	0.75	13.00	0.71
Years of practice in tennis	6.50	1.05	6.80	0.8*3*
Years of competition in tennis	3.67	0.52	3.60	0.54

The research has been developed under the recommendations of the Helsinki Declaration. Before starting the research, participants and their parents were informed about this study. Participants and their parents signed an informed consent as required by the Helsinki Declaration (2008) and the local ethics committee. The University of Extremadura Ethics Committee has also approved this research project.

### Intervention Programme

The independent variable was the tactical-cognitive training programme using video-feedback and questioning. The basis of this programme was the analysis by the players of their own decisions, by means of video-feedback and helped by the trainer with questioning. They had to visualise successful and unsuccessful decisions taken about their own actions. By viewing their actions, athletes should identify the main reasons why they took those decisions at the time. The aim of the supervisor taking part in video feedback and questioning sessions was to guide the analysis of the game situation through open questioning, but not intervening directly or giving answers to the proposed questions.

The specific characteristics of the programme were: i) supervision of 6 actions (3 successful decisions and 3 unsuccessful decisions). The order of these actions was random in every session, to prevent players from knowing if the action to be supervised was right or wrong; ii) individual analysis for every selected decision, by means of videos, showing the development of the whole point. The supervisor pointed out which action was being analysed within the whole point.

The entire set of 6 actions to be analysed comprised one complete video-feedback session. The intervention structure was similar to other intervention studies carried out on team sports [40]–[41]. Every action was supervised by means of the following structure:

Step 1. The images were viewed two consecutive times. The expert completed information with some contextual details: moment of the point in the game (e.g., fifth game of the match), scoreboard before the point (e.g., winning 3–2, score 15–40).

Step 2. Self-analysis and initial reflection of the player, where the athlete explained and initially assessed what had happened.

Step 3. Player and expert analyse together. They make a sequential analysis of the causes and reasons for the decision taken. A detailed analysis was conducted of the game context, possible solutions, decision taken, consequences and possible alternatives. All these aspects were analysed according to the tactical elements that characterise tennis shots such as: type of shot (forehand, backhand), effect (flat, topspin, backspin or slice), direction (down the line, cross-court, to the centre), depth (short, medium, long), height (high, medium or low trajectory) and hitting power. For a better understanding of the process, Table 2 shows every phase in detail, with its main aspects.

**Table 2:** Sequence for the analysis of every game action.

**STEP 1 - WATCHING selected images :**		
1.1.- Watching the full point and the selected action to be analysed.		
1.2.-Waiting moment to favour stimulated remembering.		
1.3.- New watching of the same action and contextualization of that point (scoreboard).		
Observations: The expert completes information with data such as game order in which the point is framed (e.g., fifth game of the match) and scoreboard before the beginning of the point (e.g., winning 2-1, 40-30).		
**STEP 2 – SELF-ANALYSIS and player’s reflection:**		
2.1.- Explanation and valuation of the analysed point. Global analysis of his action and initial analysis of the decision made		
**STEP 3 – COMBINED ANALYSIS player-expert:**		
Sequential analysis of the causes and reasons of the decision through questioning		
3.1.- Analysis of the decision context	Scoreboard, opponent’s placement, player’s placement, ball placement and direction, shot executed by the rival, etc. These context conditions were analysed as constraints to decision-making.	
3.2.- Valuation of possible solutions	Type of shot (forehand, backhand), effect shot (flat, topspin, backspin or slice), direction (down the line, cross-court, to the centre), depth (short, medium, long), height (high, medium or low trajectory) and shot power that might be done.Valuation of the best resources of every shot characteristic taking into account the decision context.	
3.3.- Analysis of the selected response	Type of shot (forehand, backhand), effect shot (flat, topspin, backspin or slice), direction (down the line, cross-court, to the centre), depth (short, medium, long), height (high, medium or low trajectory) and shot power selectedAnalysis of the characteristics of the shot selected respect to the decision context.	
3.4.- Analysis of the execution of the decision	Analysis of the result of the selected action. Differentiation between decision and execution.The player must differentiate between tactical intention and execution outcome.	
3.5.- Analysis of the result of the decision	Main consequences on the opponent and on the player.In a successful decision, players have to identify some good consequences (e.g., displacement of the opponent, entire space to send the next shot, etc.). In an unsuccessful decision, players have to identify some bad consequences (e.g., their displacement, loss of initiative, etc.).	
3.6.- Global analysis of the action. Proposal of alternatives (if needed)	Comparison with the analysis done in step 2.Reinforcement of decision-making or proposal of alternatives (only when analysing an error). If the decision-making analysed was successful, the player has to reinforce why the decision made was successful. If the decision-making analysed was unsuccessful, the player has to search for the best alternative after analysing context and possible solutions, and justify why this is the best.	

### Variables

The dependent variables of this study were tactical knowledge and decision-making of tennis players. By means of tactical knowledge, we purported to assess the level of cognitive expertise of these players. The analysis of tactical knowledge was conducted by means of problem representation and strategy planning [19]–[21] during a real game situation. By means of decision-making skills we want to reflect the cognitive expertise and tactical skills of athletes in real game play. Decision-making skills in tennis are defined as the adaptation of the selected shot to the specific conditions of the game situation, based on an observation instrument [24] and they are measured by the percentage of successful decision-making over the total of decisions made. This percentage was calculated for each complete match played for the pre-test (4 matches) and the post-test (4 matches) (see Figure S1).

### Instruments

**Tactical knowledge.** The verbal protocol validated for tennis in previous studies [6], [13], [19]–[21], [42] was used to evaluate tactical knowledge as a reflection of cognitive expertise.

This instrument, which measures tactical knowledge, consists of carrying out interviews during the action, and then coding this interview by means of a system of categories structured into 3 levels (conceptual content, conceptual sophistication and conceptual structure), both in problem representation and in strategy planning.

Interview procedures: the interview comprises two questions developed to access the problem representation and the strategy planning of tennis players, following the very same procedure as in other similar studies [6], [21], [42]. Interviews were collected during a real game situation and they were carried out on the actual court where players were competing, during a full set. Prior studies indicated that the athletes’ performance was not affected by the application of interviews [24]. Before beginning this process, participants were familiarised with the data collection situation. The researcher explained to players that they did not have any time limitation to answer and they had to answer the questions asked as accurately as they could, reporting the information about previous and current thoughts. Two previous interviews were carried out following the explanation and before the start of the measurement in order to verify that the athletes responded accurately and with no time constraint. After that, they carried out a 10-minute warm-up before starting the game, as established by the Spanish Tennis Federation rules. During the pauses between two points, players answered two questions. The first question of the verbal protocol was asked immediately after ending every point: *‘What were you thinking about while playing this point?’* (Recall Interview-RI [6], [42]). This question requires players to remember their thoughts about the previous point. Verbalisations made with this question will assess problem representation. The second question is: *‘What are you thinking about now?’* (Planning Interview-PI [6], [21]). This was asked immediately after the RI and immediately before playing the following point. By means of this question, players inform about their current thoughts, possibilities about subsequent points, and also assess their planning. Answers obtained in this case assess the strategy planning. Likewise, this procedural knowledge assessment method is developed in performance environments where athletes have to respond to real competition situations, which permits enhancing the validity of the data [43].

Coding system: 16 interviews were selected at random for every individual from the total number of interviews carried out in a real game situation, following the same criteria as in previous research studies [6]. After transcribing the interviews, these two questions were separated. Within the coding process, they were firstly divided into concepts or information units, and later they were coded by means of a system of categories into three levels of analysis. The first level of analysis is *Conceptual Content* (comprised of *Main concept categories* and *Subconceptual categories*). The second level of analysis is *Conceptual Sophistication*, and the third level of analysis is *Conceptual Structure* (see [6] for a review).

Within the quantitative analysis of verbal reports, in order to analyse the first level (conceptual content: total and variety of concepts), the total value as well as of a variety of each one of the main conceptual categories (goal, condition, action, and regulatory concepts) was obtained. For conceptual sophistication (level 2) all the verbalised concepts were analysed at all the levels of goals, conditions and actions concepts. The conceptual structure (level 3) was assessed by means of the total number of linkages and the total number of structures found (double and triple).

Reliability of the coding system: in order to code interviews, only one coder was used, who underwent coding training taught by one of the researchers. Coder had a Sport Sciences Degree, specific training as a tennis coach (Level 2 in the Spanish Tennis Federation), and also specific training in coding. Reliability was calculated for all three levels of analysis in every question (RI and PI). To obtain the required coding reliability, 10 training sessions were held sequentially with the coder and at different moments in time. Reliability between coders (researcher-coder) was calculated using Cohen’s Kappa index, obtaining values of up to.75 [44] to ensure reliability. Temporal reliability was also ensured, coding the same interviews 10 days later with a test-retest reliability of up to.80.

**Decision-making.** To assess decision-making in tennis players, an observation instrument was used to test tactical skills in real game play [24]. This has already been used for other studies, applied to tennis players with different expertise [24], [42], [45]. The decision-making section on “game play” taken from this instrument, which assesses all shots made after serve, was used. This is a useful instrument to assess expertise and performance differences, complying with the recommendation of Travassos et al. [43] about the need to perform sporting actions under *in situ* conditions to assess expertise effect.

The instrument evaluates decision-making skills in every action as ‘successful decisions’ (value 1) and ‘unsuccessful decisions’ (value 0), by means of criteria that assess the player’s intention (offensive or defensive) according to: opponent’s position, player’s position, ball trajectory, and the intention of putting pressure on the opponent (trying to displace the opponent, keeping him/her on the baseline, playing to his/her weak side, etc.) or defending (recovering position after a forceful shot by the opponent) [24].

Decision-making skills were assessed by means of the percentage of successful decisions (value 1) made during the match. Filming and observation conditions proposed by Nielsen and McPherson [24] were taken into account in order to apply the observation instrument.

Inter- and intra- observer’s reliability: To correctly observe all the matches of every player, 2 experienced observers were selected. Observers were external to the study, they had a Sport Sciences Degree, with specific training as tennis coaches (Level 2 in the Spanish Tennis Federation) with at least five years’ experience as coaches at this level, and also specific training in systematic observation processes through a Master Course. Cohen’s Kappa statistic was calculated for inter- and intra- observers reliability. Values achieved were higher than.81 in inter-observers’ reliability, and higher than.84 for intra-observers’ reliability in all observers. Values over.75 establish very good/almost complete concordance [44].

### Procedures

In order to carry out this study, a quasi-experimental design was developed with two groups: control and experimental. A total of 18 matches were carried out for each tennis player. Tactical knowledge was measured before and after applying the intervention programme (pre-test and post-test), selecting 16 random interviews out of all the interviews held for every individual participating in this study. Decision-making measures were assessed during 4 matches before (pre-test) and 4 matches after (post-test) applying the intervention programme (see Figure S1).

As shown in Figure S1, the 18 matches played by all the players were distributed in three phases. The first 4 matches were used as a pre-test of the decision-making variable. After these 4 matches, the pre-test of the tactical knowledge was carried out (problem representation and strategy planning) in a real game situation, as explained above. After performing the pre-test of all the variables, the players were divided into two groups (experimental and control). The following 10 matches (from match 5 to match 14) the intervention was carried out with the experimental group, developing the intervention programme based on video-feedback and questioning for 10 sessions after each one of the matches. All the players developed the same number of matches and training sessions. Finally, the final 4 matches (from match 15 to match 18) were used to make the post-test measurement of the decision-making variable. The post-test of tactical knowledge (problem representation and strategy planning) was carried out after the 18 matches.

Furthermore, during the entire time the research lasted, the athletes did their on-court training as usual, regardless of the group they belonged to. This training lasted for 4 hours a week spread out over 3 days. The athletes from the control group and from the experimental group accumulated a total of 72 hours’ training throughout the entire research. All players trained at the same time and with the same coach. The eleven players competed in 18 matches (1 match per week) during the research. Only the players from the experimental group received the intervention programme explained above, which lasted for a maximum of 40 minutes per session and per player. Thus, the tennis players in the control group accumulated 72 hours’ training and 18 competition matches, and the tennis players from the experimental group accumulated 72 hours’ training, 18 competition matches and a maximum of 7 hours’ intervention programme.

To obtain a competition structure that would guarantee a continuous weekly intervention by means of video-feedback and questioning, and on a real game situation, we developed a round-robin structure, in which each player meets all other players in turn. They had to play one match every week. By means of this system, it was possible to play 18 matches on a continuous basis. Thus, the experimental group could carry out 10 video-feedback sessions, one after every match played.

Matches were played on Sundays. The video-feedback programme was applied the following day, within 24 hours after competition and before the first training session of the week.

The on-court trainer was not informed about the specific objectives of the programme in order not to influence this process.

### Data analysis

Results were obtained by parametric and non-parametric statistics because the values were achieved by means of the Shapiro-Wilks test (for samples with less than 30 individuals) indicating data normality in decision-making and non-normality in tactical knowledge variables.

Mean and standard deviation was used to describe variables, and also mean ranks were reported to tactical knowledge variables. To analyse the effect of the intervention programme four 2×2 Mixed MANOVAs (Test-time x Group) were used for tactical knowledge variables, following the procedures established by Thomas, Nelson & Thomas [46] and Thomas & Thomas [47] to use multivariate analyses with non-parametric variables. These variables were grouped to perform MANOVAs into: i) Concept content-total; ii) Concept content-variety; iii) Concept Sophistication; and iv) Concept Structure. In 2×2 Mixed MANOVAs, a repeated measure factor (pre-test and post-test) and a Group factor (Experimental and Control group) were used. Later, univariate ANOVAs were reported to test differences in every tactical knowledge variable. These analyses follow the method of Puri & Sen [48] Rank-Order General Linear Method. This method maintains good power and protects against type I errors, having to develop three steps: a) change data to ranks (mean ranks reported in descriptive statistics); b) use standard parametric procedures with ranked scores; c) calculate the *L-*statistic instead of *F* [46]-[47], [49].

The *L*-statistic for all MANOVAs  =  [(*N* – 1) *r* ^2^], where *r* ^2^  =  Pillai’s trace. Univariate analyses of variances (ANOVAs) were conducted to individually test differences in variables. The *L*-statistic for all *post-hoc* ANOVAs  =  [(*N – 1) r* ^2^], where *r* ^2^ = *SS*_Between_/*SS*_Total_. All the MANOVAs performed meet the requirements and assumptions of MANOVA designs about normality (in ranked data) and multicollinearity. For repeated measures, the sphericity assumption was met in all variables, with a value of 1.0 in the Huynh-Feldt epsilon [46]. These non-parametric procedures have been used in other studies to test differences in tactical knowledge in tennis (e.g., [6]).

In addition, a 2×2 Mixed ANOVA (Test-time x Group) was conducted to assess the effect of the intervention programme on the decision-making variable, using a repeated measures factor (Test-time: pre-test and post-test) and a Group factor (Experimental and Control). The estimation of the Effect Size was included in Mixed ANOVA for decision-making, by means of partial squared Eta (η_p_^2^) to improve the assessment of the differences found, because it eliminates the influence of the sample size.

Alpha was set at 0.05, and 95% Confidence Interval for differences (Diff. 95% CI) was reported. The IBM SPSS Statistics v.19.0 statistical program was used for data analysis.

## Results

### Problem representation

To test the effect of the intervention programme on problem representation, a 2×2 Mixed MANOVA was performed on Concept Content-total, Concept Content-variety, Concept Sophistication and Concept Structure. In each Mixed MANOVA, multivariate contrasts were reported and univariate contrasts were included in Tables 3 and 4 to show variables differences (see also Appendix S1).

**Table 3:** Mean, standard deviation, mean rank and univariate contrast in problem representation variables for pre–test.

Variables	Control group (*n* = 6)			Experimental group (*n* = 5)			L_(1)_	*p*	Diff. 95% CI [LO,HI]
	M	SD	MR	M	SD	MR			
*Concept Content (total)*									
Total concepts	43.33	11.74	5.50	45.60	6.23	6.60	0.30	>.05	[–5.8,3.6]
Total goals	18.33	8.26	5.83	17.60	7.92	6.20	0.03	>.05	[–5.1,4.4]
Total conditions	10.50	8.07	5.17	12.80	6.91	7.00	0.84	>.05	[–6.4,2.7]
Total actions	11.67	5.46	6.58	9.20	4.21	5.30	0.41	>.05	[–3.4,5.9]
Total regulatory	2.67	2.07	5.00	4.80	3.11	7.20	1.23	>.05	[–6.6,2.2]
*Concept Content (variety)*									
Variety goals	6.17	1.33	6.83	5.20	1.64	5.00	0.93	>.05	[–2.5,6.2]
Variety conditions	4.83	2.86	5.33	5.80	1.48	6.80	0.54	>.05	[–6.1,3.2]
Variety actions	4.50	1.64	6.50	4.20	1.09	5.40	0.33	>.05	[–3.4,5.6]
Variety regulatory	1.67	1.03	4.83	2.60	0.89	7.40	1.95	>.05	[–6.5,1.4]
*Concept sophistication*									
*Goal hierarchies*									
1-Selves & opponent	5.50	4.76	4.83	9.80	6.14	7.40	1.64	>.05	[–6.9,1.8]
2-Win attributes	3.17	1.72	6.58	2.60	1.34	5.30	0.44	>.05	[–3.2,5.8]
*Condition qualities*									
2-Appropriate-one feature	6.33	4.41	6.25	5.80	3.27	5.70	0.08	>.05	[–4.2,5.3]
3-Appropriate-two features	1.67	1.51	6.00	1.60	1.52	6.00	0.00	>.05	[–4.7,4.7]
*Action qualities*									
2-Appropriate-one feature	6.50	2.88	6.33	5.00	2.91	5.60	0.14	>.05	[–4.0,5.5]
3-Appropriate-two features	2.33	2.25	6.08	2.00	1.58	5.90	0.01	>.05	[–4.5,4.9]
*Concept structure*									
N° linkages	22.50	8.45	5.25	25.20	6.91	6.90	0.68	>.05	[–6.2,2.9]
Double-concept linkages	7.67	1.97	6.25	1.64	13.5	5.70	0.08	>.05	[–4.1,5.2]
Triple-concept linkages	5.00	2.28	5.25	1.82	10.5	6.90	0.70	>.05	[–6.2,2.9]

MR  =  Mean Rank.

**Table 4:** Mean, standard deviation, mean rank and univariate contrasts in problem representation variables for post–test.

Variables	Control group (*n* = 6)			Experimental group (*n* = 5)			L_(1)_	*p*	Diff. 95% CI [LO,HI]
	M	SD	MR	M	SD	MR			
*Concept Content (total)*									
Total concepts	38.83	8.97	3.50	75.80	25.81	9.00	7.53	**<.01**	[–7.9, –3.1]
Total goals	15.83	7.00	4.50	25.80	10.23	7.80	2.71	>.05	[–7.4,0.8]
Total conditions	9.00	7.72	4.42	24.00	16.98	7.90	3.04	>.05	[–7.5,0.5]
Total actions	10.17	3.71	4.50	16.60	6.69	7.80	2.76	>.05	[–7.3,0.7]
Total regulatory	2.50	2.81	4.17	8.20	5.54	8.20	4.11	**<.05**	[–7.7, –0.4]
*Concept Content (variety)*									
Variety goals	5.50	1.38	4.75	7.20	1.79	7.50	1.98	>.05	[–6.9,1.4]
Variety conditions	4.67	3.72	4.75	7.80	2.28	7.50	1.90	>.05	[–7.0,1.5]
Variety actions	4.50	2.07	5.25	5.60	1.14	6.90	0.71	>.05	[–6.1,2.8]
Variety regulatory	1.67	1.51	4.17	3.80	1.30	8.20	4.17	**<.05**	[–7.6, –0.4]
*Concept sophistication*									
*Goal hierarchies*									
1-Selves & opponent	6.00	4.00	3.92	13.40	3.85	8.50	5.28	**<.05**	[–7.8, –1.3]
2-Win attributes	3.00	2.37	5.67	3.20	1.64	6.40	0.14	>.05	[–5.4,3.9]
*Condition qualities*									
2-Appropriate-one feature	5.83	5.11	4.92	10.20	7.22	7.30	1.45	>.05	[–6.7,1.9]
3-Appropriate-two features	1.50	1.97	3.67	9.20	6.42	8.80	6.81	**<.01**	[–7.8, –2.5]
*Action qualities*									
2-Appropriate-one feature	6.67	2.34	6.17	7.20	4.76	5.80	0.03	>.05	[–4.3,5.0]
3-Appropriate-two features	1.50	1.05	3.58	7.40	3.21	8.90	7.11	**<.01**	[–7.9,2.8]
*Concept structure*									
N° linkages	20.50	4.09	3.50	47.80	17.30	9.00	7.57	**<.01**	[–7.8,,3.1]
Double-concept linkages	8.50	2.07	7.58	5.80	2.39	4.10	3.04	>.05	[–0.5,7.5]
Triple-concept linkages	4.67	2.25	3.50	12.60	2.88	9.00	7.64	**<.01**	[–7.8, –3.2]

MR  =  Mean Rank.

There was no significant main effect for Group in Concept Content-total variables interaction in pre-test (L(3)  = 2.15; p >.05; see Table 3). In contrast, in post-test, there was a significant main effect for Group in Concept Content-total variables interaction (L(3)  = 8.74; p <.05). Univariate follow-up analyses of Concept Content-total variables in post-test (Table 4) show significant differences between experimental and control group in Total of concepts (p <.01) and Total regulatory concepts (p <.05) with higher values in the experimental group.

No significant main effect was found for Group in Concept Content-variety variables interaction in pre-test (L(3)  = 2.47; p >.05; see Table 3). In post-test, there was no significant main effect, either, of Concept Content-variety variables (L(3)  = 6.36; p >.05; see Table 4).

No significant main effect was found for Group in Concept Sophistication variables interaction in pre-test (L(3)  = 6.20; p >.05; see Table 3). In contrast, in post-test, there was a significant main effect for Group in Concept Sophistication variables interaction (L(3)  = 9.35; p <.05). Follow-up univariate analysis of Concept Sophistication variables in post-test (Table 4) show significant differences between experimental and control group in Goal Concepts Level 2 (p <.05), Condition Concepts Level 3 (p <.01) and Action Concepts Level 3 (p <.01) with higher values in the experimental group.

The MANOVA for Concept Structure showed no significant main effect for Group in Concept Structure variables interaction in pre-test (L(2)  = 0.75; p >.05; see Table 3). In contrast, in post-test, there was a significant main effect for Group in Concept Structure variables interaction (L(2)  = 7.79; p <.05). Follow-up univariate analysis of Concept Structure variables in post-test (Table 4) showed significant differences between experimental and control group in number of linkages (p <.01) and triple concepts (p <.01) with higher values in the experimental group.

### Planning strategy

The same analyses were performed in planning strategy. Multivariate contrasts for each Mixed MANOVA were reported, and univariate contrasts were included in Tables 5 and 6 to show variables differences (see also Appendix S1).

**Table 5:** Mean, standard deviation, mean rank and univariate contrast in planning strategy variables for pre–test.

Variables	Control group (*n* = 6)			Experimental group (*n* = 5)			L_(1)_	*p*	Diff. 95% CI [LO,HI]
	M	SD	MR	M	SD	MR			
*Concept Content (total)*									
Total concepts	36.50	7.29	6.75	34.00	4.30	5.10	0.68	>.05	[–2.9,6.2]
Total goals	20.17	4.71	6.17	19.40	7.89	5.80	0.03	>.05	[–4.4,5.1]
Total conditions	7.83	5.46	6.75	5.80	4.09	5.10	0.70	>.05	[–2.9,6.1]
Total actions	8.00	3.85	7.17	5.60	3.65	4.60	1.69	>.05	[–1.7,6.9]
Total regulatory	1.00	1.26	6.25	0.80	1.09	5.70	0.09	>.05	[–3.8,4.9]
*Concept Content (variety)*									
Variety goals	6.67	1.86	6.33	6.20	1.48	5.60	0.14	>.05	[–3.9,5.4]
Variety conditions	3.50	2.26	6.42	3.20	1.64	5.50	0.21	>.05	[–3.8,5.6]
Variety actions	4.17	1.83	6.92	2.80	1.92	4.90	1.05	>.05	[–2.4,6.5]
Variety regulatory	0.83	0.98	6.58	0.40	.55	5.30	0.50	>.05	[–2.9,5.5]
*Concept sophistication*									
*Goal hierarchies*									
1-Selves & opponent	5.50	4.18	5.00	8.00	2.74	7.20	1.23	>.05	[–6.6,2.2]
2-Win attributes	4.83	2.64	6.17	4.40	2.70	5.80	0.03	>.05	[–4.3,5.1]
*Condition qualities*									
2-Appropriate-one feature	3.50	2.17	7.17	2.20	.84	4.60	1.68	>.05	[–1.7,6.9]
3-Appropriate-two features	1.17	1.17	6.17	1.00	1.00	5.80	0.04	>.05	[–4.2,4.9]
*Action qualities*									
2-Appropriate-one feature	4.50	1.64	6.92	3.20	1.79	4.90	1.07	>.05	[–2.4,6.4]
3-Appropriate-two features	0.67	0.82	6.00	0.60	0.55	6.00	0.00	>.05	[–4.3,4.3]
*Concept structure*									
N° linkages	17.33	4.55	6.92	15.20	3.27	4.90	1.01	>.05	[–2.5,6.5]
Double-concept linkages	7.17	1.60	6.42	6.60	1.52	5.50	0.22	>.05	[–3.7,5.5]
Triple-concept linkages	4.33	2.66	6.58	3.80	1.64	5.30	0.42	>.05	[–3.3,5.9]

MR  =  Mean Rank.

**Table 6:** Mean, standard deviation, mean rank and univariate contrast in planning strategy variables for post–test.

Variables	Control group (*n* = 6)			Experimental group (*n* = 5)			L_(1)_	*p*	Diff. 95% CI [LO,HI]
	M	SD	MR	M	SD	MR			
*Concept Content (total)*									
Total concepts	35.50	8.41	4.00	51.80	9.20	8.40	**4.80**	**<.05**	[–7.9, –0.9]
Total goals	20.50	4.32	4.42	29.00	10.30	7.90	3.02	>.05	[–7.4,0.5]
Total conditions	6.17	5.64	5.00	10.00	2.12	7.20	1.22	>.05	[–6.7,2.3]
Total actions	8.50	2.88	4.92	11.80	4.60	7.30	1.44	>.05	[–6.8,2.0]
Total regulatory	0.17	0.41	5.83	0.40	0.89	6.20	0.07	>.05	[–3.6,2.8]
*Concept Content (variety)*									
Variety goals	6.83	1.17	5.00	7.80	1.64	7.20	1.26	>.05	[–6.6,2.2]
Variety conditions	3.50	2.88	4.92	5.20	2.59	7.30	1.43	>.05	[–6.8,2.0]
Variety actions	4.00	1.09	6.75	3.40	1.14	5.10	0.74	>.05	[–2.7,6.0]
Variety regulatory	0.17	0.41	5.92	0.20	0.45	6.10	0.02	>.05	[–3.4,3.0]
*Concept sophistication*									
*Goal hierarchies*									
1-Selves & opponent	7.83	3.06	4.17	14.00	5.61	8.20	**4.11**	**<.05**	[–7.6, –0.4]
2-Win attributes	4.17	2.48	7.00	2.60	1.14	4.80	1.25	>.05	[–2.2,6.6]
*Condition qualities*									
2-Appropriate-one feature	3.67	3.33	5.33	4.80	2.28	6.80	0.56	>.05	[–6.0,3.1]
3-Appropriate-two features	0.67	1.21	4.42	1.80	0.84	7.90	3.24	>.05	[–7.3,0.3]
*Action qualities*									
2-Appropriate-one feature	6.00	2.37	6.17	6.20	3.35	5.80	0.03	>.05	[–4.3,5.1]
3-Appropriate-two features	1.00	1.09	3.67	5.60	2.88	8.80	**8.61**	**<.01**	[–6.7, –3.6]
*Concept structure*									
N° linkages	17.50	4.76	3.83	29.60	5.18	8.60	**5.66**	**<.05**	[–7.9, –1.6]
Double-concept linkages	8.50	2.95	6.83	6.80	3.42	5.00	0.84	>.05	[–2.7,6.4]
Triple-concept linkages	3.33	2.42	3.58	7.80	1.09	8.90	**7.20**	**<.01**	[–7.8, –2.8]

MR  =  Mean Rank.

There was no significant main effect for Group in Concept Content-total variables interaction in pre-test (L(3)  = 5.25; p >.05; see Table 5). In post-test, there was no significant main effect for Group, either, of Concept Content-total variables interaction (L(3)  = 5.75; p >.05; see Table 6).

No significant main effect was found for Group in Concept Content-variety variables interaction in pre-test (L(3)  = 3.38; p >.05; see Table 5). In post-test, there was no significant main effect for Group, either, of Concept Content-variety variables interaction (L(3)  = 3.49; p >.05; see Table 6).

The MANOVA showed no significant main effect for Group in Concept Sophistication variables interaction in pre-test (L(3)  = 6.78; p >.05, see Table 5). In contrast, in post-test, there was a significant main effect for Group in Concept Sophistication variables interaction (L(3)  = 9.25; p <.05). Follow-up univariate analysis of Concept Sophistication variables in post-test (Table 6) showed significant differences between experimental and control groups in Goal Concepts Level 2 (p <.05) and Action Concepts Level 3 (p <.01) with higher values in the experimental group.

No significant main effect was found for Group in Concept Structure variables interaction in pre-test (L(2)  = 1.27; p >.05; see Table 5). In contrast, in post-test, there was a significant main effect for Group in Concept Structure variables interaction (L(2)  = 7.23; p <.05). Follow-up univariate analysis of Concept Structure variables in post-test (Table 6) showed significant differences between experimental and control groups in number of linkages (p <.05) and triple concepts (p <.01) with higher values in the experimental group.

### Decision-making

For decision making, a 2×2 Mixed ANOVA was conducted out. Multivariate analysis shows a significant effect in the interaction of Test-time and Group (Wilks Lambda  = .318; F(1,9)  = 19.318; p  = .002; η_p_^2^ = .682). Univariate analysis showed no significant differences between experimental and control groups in pre-test (F(1,9)  = 2.261; p = .167; η_p_^2^ = .201). In Post-test there was a significant difference between experimental and control groups (F(1,9)  = 6.017; p = .037; η_p_^2^ = .401) with significant higher values of decision-making in the experimental group (Table 7 and Appendix S1).

**Table 7:** Mean, standard deviation and differences analysis in decision-making.

Test-time	Control Group (*n* = 6)		Experimental Group (*n* = 5)		Mean diff.	Typical Error	F _(1,9)_	*p* *	η_p_^2^	Diff 95% CI [LO,HI]
	M	SD	M	SD						
Pre-test	58.65	2.46	55.54	4.31	3.10	2.06	2.26	.167	.201	[–1.5,7.7]
Post-test	54.95	4.25	61.20	4.15	–6.25	2.55	6.01	**.037**	**.401**	[–12.0, –0.5]

^*^ Bonferroni adjust for multiple comparisons.

## Discussion

The hypothesis of our study suggested that the application of a tactical-cognitive training programme based on video-feedback and questioning would have a positive effect on the tactical knowledge of tennis players, in content, sophistication and structure. Moreover, an improvement of athletes’ cognitive expertise will produce an increase of decision-making skills with a higher percentage of successful decision-making in real game play.

Effects produced on conceptual content show that in both problem representation and strategy planning, players who took part in the video-feedback and questioning programme have modified this conceptual content, verbalising more concepts. Experimental group players get closer to an expert profile, with greater knowledge, where approaches to sport situations carried out in a global manner (typical of a lower level of expertise) are being substituted by more tactical approaches, producing a greater number of adaptations in LTM [6], [22].

In the experimental group, significant differences with a higher quantity of regulatory concepts in problem representation show a greater and more varied evaluation of their actions and greater self-evaluation capacity, related to players with a higher level of expertise [19], [50]. These regulatory concepts, already developed, will help increase athletes’ comprehension of factors that have an influence on their performance [9] and also help to make more accurate decisions [24].

With reference to conceptual sophistication, it is possible to find a significantly larger number of goals related to the opponent, both in problem representation and in strategy planning. The greater number of references to the opponent in verbalisations shows that there is a development of tactical knowledge and cognitive skills related to higher levels of expertise, by means of tactics related to the opponent, and permitting a high specialisation of cognitive skills [12], [51].

In condition sophistication, the experimental group develops a significantly larger number of concepts with two or more features, both in problem representation and in strategy planning. In quality of actions (level 3), these significant differences can only be found in strategy planning. Once again, tennis players from the experimental group get closer to the expert profile by developing more sophisticated concepts, because a more detailed problem representation is a characteristic feature of expert performance, especially in condition and action concept quality [9], [22], [52]–[53]. Thus, there is a more in-depth interpretation and with more tactical aspects, based on evaluations about the opponent, tendencies or more detailed weakness [13], [53]. On the other hand, less concept sophistication in the control group shows that they continue to make more global approaches to sport situations with less interpretation of the environment [53], whilst the experimental group shows signs of change, from a superficial processing of the environment (related to lower levels of expertise) towards deep and tactical processing [13], [54].

Within the conceptual structure, similar changes have been achieved, as the total number of connections, and the number of triple verbalised concepts, both in problem representation and in strategy planning by the experimental group is significantly higher. Results show once again that these players get closer to the expert profile, developing a greater interrelation of knowledge and concept association, and with a structural complexity that is more typical of players with greater expertise [9], [20]. Likewise, this greater structuring of knowledge shows how concepts unite to form more complex profiles that are developed in the LTM and reflect a higher level of expertise, because these adaptations and improvements, exemplified in greater structures of the LTM, will favour better decisions [6], [12]–[13].

Differences found here enable us to observe how experimental group players developed a higher problem representation, where the use of tactical knowledge is greater and will be more effective when trying to solve a game situation. They also developed a greater, more elaborated and more sophisticated, strategy planning, which will be used to plan subsequent actions based on relevant information for different game situations [6], [13], [19]–[21]. These adaptations will enable us to carry out continuous and dynamic coding that will favour the athlete in order to anticipate events, to predict results and to develop proactive behaviours [17].

After the tactical-cognitive training programme, and when they get closer to the expert profile, the experimental group players make more successful decisions.

These improvements in decision-making skills show a higher quality of decision-making because the use of specific strategies enables them to take better decisions and make strategically better selections [24]. This improvement is due to the fact that the decisions made will take into account a larger number of conditions produced in the game context (i.e.: court position, opponent’s position, or the scoreboard), as it reflects the results in tactical knowledge. They will also interpret tendencies, strengths and weaknesses in a more sophisticated manner, taking their opponents into account as well as their own features. And furthermore, they will take into account the different contextual conditions that may have an influence on shot selection [19], [22], [24].

Our results also show how the knowledge base of those players who have been exposed to the tactical-cognitive training programme based on video-feedback and questioning have evolved towards characteristics typical of a higher level of expertise, where: conditions and weak actions change into tactical and associated concepts; global approaches to sporting situations turn into tactical approaches with the presence of relevant information; and where the superficial processing of events of the environment is replaced with a deep processing of information [13], [26].

The development of adaptations in the LTM such as current event profiles and action plan profiles require several years of experience [6], [12], but by means of formative strategies such as the video-feedback and questioning programme, it can be developed faster and more effectively. These profiles will enable players to code, update and modify the relevant conditions for decision-making in the competition [55], [56]. The development of these adaptations in the LTM also explains these improvements in decision-making. The video feedback and questioning tools develop adaptations in the action plan profiles and current event profiles. This improvement of tactical knowledge will permit the improvement of decision-making capacities because knowledge (i.e., problem representations) guides decision-making [9], [45].

It is critical for athletes to gain an intellectual understanding of the implications of their behaviour to develop their decision-making skills and to get opportunities to practice these new skills [11]. An explicit learning strategy, like the programme shown in this work, could be effective for highly complex situations (like the ones that occur in tennis), and that are related to deliberate decision-making [35], [36].

Furthermore, developing cognitive processes for deliberate decision-making may be the basis for processing information faster and using intuitive processes [11], [57], as well as for prioritising information in situations involving time pressure and choosing the best option via simple heuristics [58]. Time pressure can determine which decisions are taken via deliberate processes and which are taken via intuitive and heuristic processes (e.g., “Take-the-first”), as intuitive and deliberate processes seem to be used in the various stages of the decision-making process in sports [36].

Therefore, this type of decisional training would not only be useful for deliberate decision-making but it may also be the basis of intuitive decision-making. Furthermore, when the expertise level increases, the time pressure to take decisions is also greater for athletes [59]. In this sense, the level of expertise may be a decisive factor to choose the most adequate decisional training processes, as explicit decisional learning processes may be more effective at lower levels of expertise, whilst implicit decisional learning processes (e.g., based on ecological dynamics and manipulation of task constraints; for a review in tennis, see [60]) may be more effective for higher levels of expertise.

With reference to the application of similar programmes, results of this study set out the effectiveness of tactical-cognitive training based on video-feedback and questioning on several cognitive variables, in the same way as in other studies developed in team sports [40], [41]. It is also possible to confirm the effectiveness of video-feedback and questioning as useful tools for cognitive training, together with the help of an expert or supervisor to direct attention towards some signs [31], [34]. It would be interesting to consider future studies with placebo group, or applying these tools separately, where the contribution of each tool to decisional learning can be assessed more directly, or if really by combining video-feedback and questioning greater effects are obtained.

One of the limitations of the study is the small number of participants, which limits the capacity to extrapolate the results. However, the reduced number of individuals has enabled us to study both the tactical knowledge and decision-making in tennis players in greater depth and detail, as well as to try to identify the underlying processes in cognitive expertise and in their training process. On the other hand, the need is raised to continue investigating into the relationships between cognitive variables and performance variables, both in tennis and in other sports.

Finally, we would like to highlight the effect produced on different variables related to cognitive expertise and decision-making, and the need to promote programmes focused on improving cognitive areas (by means of video-feedback, questioning or by some other tactical-decisional training tools) because of their importance during athletes’ formative stages and the very strong relationship between cognition and performance in sport. To this end, it would be important for future studies to incorporate variables that may be direct performance indicators (e.g. through notational analysis) and verify the effect of decisional training on performance.

## Supporting Information

Figure S1 **Overall procedure and research design.** Distribution of the 18 matches played, decision-making pre- and post-test, tactical knowledge pre- and post-test, on-court sessions in both groups, and intervention sessions in experimental group.
(JPG)

Appendix S1 **Value graphics of variables with significant differences.** **Graphic 1.** Values of percentage of decision-making for pre- and post-test in each player. DM  =  Decision-making; C1 = Control Player 1; C2 = Control Player 2; C3 = Control Player 3; C4 = Control Player 4; C5 = Control Player 5; C6 = Control Player 6; E1 = Experimental Player 1; E2 = Experimental Player 2; E3 = Experimental Player 3; E4 = Experimental Player 4; E5 = Experimental Player 5. **Graphic 2.** Values of total concepts in problem representation for pre- and post-test in each player. Tot Concept  =  Total of concepts; PR = Problem Representation; Abbreviations in X-axis are described in Graphic 1. **Graphic 3.** Values of total regulatory concepts in problem representation for pre- and post-test in each player. Tot Reg  =  Total regulatory concepts; PR = Problem Representation; Abbreviations in X-axis are described in Graphic 1. **Graphic 4.** Values of variety of regulatory concepts in problem representation for pre- and post-test in each player. Var Reg = Variety of regulatory concepts; PR = Problem Representation; Abbreviations in X-axis are described in Graphic 1. **Graphic 5.** Values of goal level 1 concepts in problem representation for pre- and post-test in each player. Goal-1 = Goal Level 1 concepts; PR = Problem Representation; Abbreviations in X-axis are described in Graphic 1. **Graphic 6.** Values of condition level 3 concepts in problem representation for pre- and post-test in each player. Cond-3 = Condition Level 3 concepts; PR = Problem Representation; Abbreviations in X-axis are described in Graphic 1. **Graphic 7.** Values of action level 3 concepts in problem representation for pre- and post-test in each player. Act-3 = Action Level 3 concepts; PR = Problem Representation; Abbreviations in X-axis are described in Graphic 1. **Graphic 8**. Values of number of linkages in problem representation for pre- and post-test in each player. PR = Problem Representation; Abbreviations in X-axis are described in Graphic 1. **Graphic 9.** Values of triple concepts in problem representation for pre- and post-test in each player. Triple = Triple concepts; PR = Problem Representation; Abbreviations in X-axis are described in Graphic 1. **Graphic 10.** Values of total concepts in planning strategy for pre- and post-test in each player. Tot Concept = Total Concepts; PS = Planning strategy; Abbreviations in X-axis are described in Graphic 1. **Graphic 11.** Values of goal level 1 concepts in planning strategy for pre- and post-test in each player. Goal-1 = Goal Level 1; PS = Planning strategy; Abbreviations in X-axis are described in Graphic 1. **Graphic 12.** Values of action level 3 concepts in planning strategy for pre- and post-test in each player. Act-3 = Action Level 3 concepts; PS = Planning strategy; Abbreviations in X-axis are described in Graphic 1. **Graphic 13.** Values of number of linkages in planning strategy for pre- and post-test in each player. PS = Planning strategy; Abbreviations in X-axis are described in Graphic 1. **Graphic 14.** Values of triple concepts in planning strategy for pre- and post-test in each player. Triple = Triple concepts; PS = Planning strategy; Abbreviations in X-axis are described in Graphic 1.
(DOC)
