# Supplementary figures and images for: Effectiveness of a Video-Feedback and Questioning Programme to Develop Cognitive Expertise in Sport
Source: PLoS One. 2013 Dec 10;8(12):e82270. doi: 10.1371/journal.pone.0082270 (PMC3858278; doi:10.1371/journal.pone.0082270)

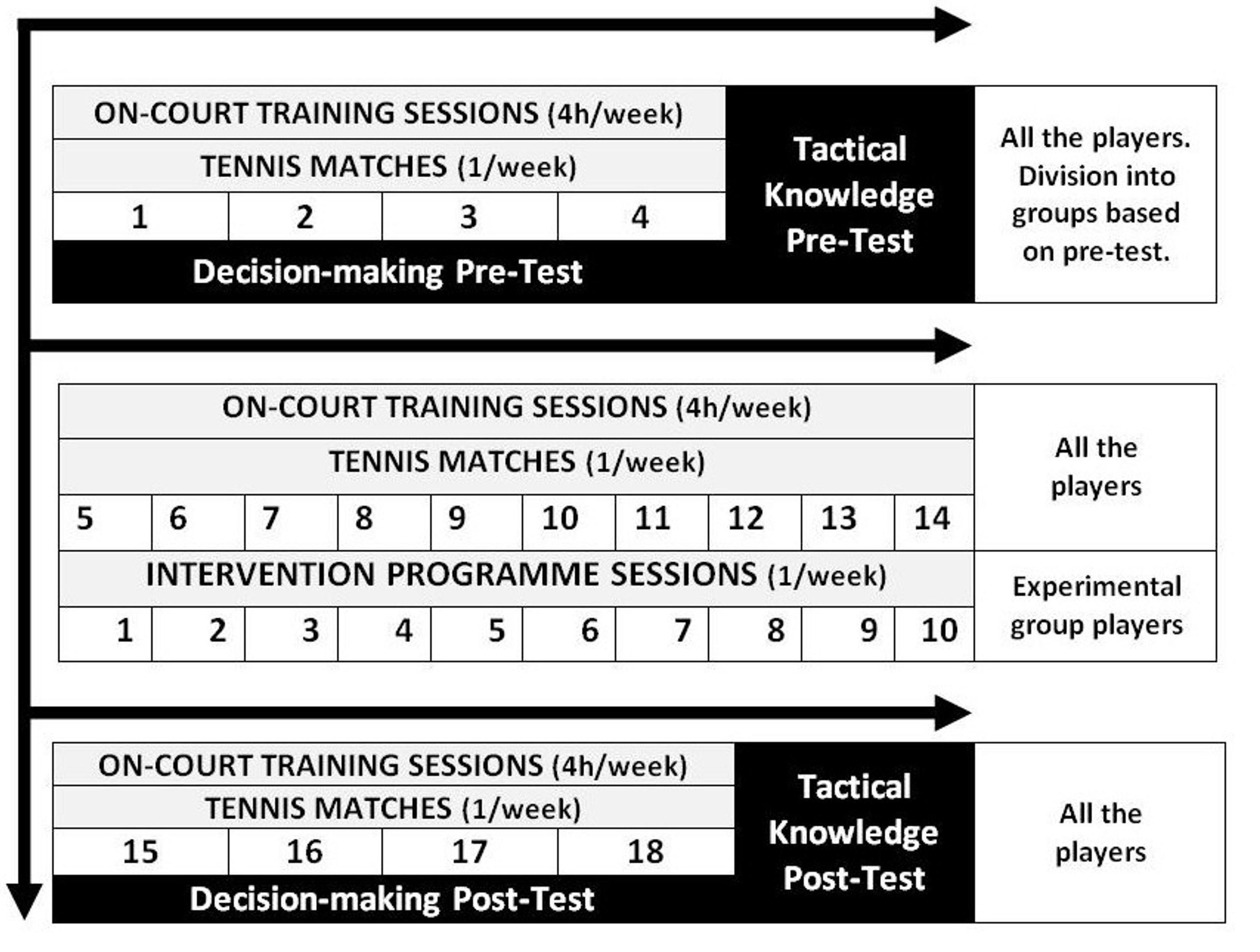

Supplement: Figure S1 — Overall procedure and research design. Distribution of the 18 matches played, decision-making pre- and post-test, tactical knowledge pre- and post-test, on-court sessions in both groups, and intervention sessions in experimental group. (JPG) [file pone.0082270.s001.jpg]
