# Supplementary material for: Effectiveness of a Video-Feedback and Questioning Programme to Develop Cognitive Expertise in Sport
Source: PLoS One. 2013 Dec 10;8(12):e82270. doi: 10.1371/journal.pone.0082270 (PMC3858278; doi:10.1371/journal.pone.0082270)
Supplement: Appendix S1 — Value graphics of variables with significant differences. Graphic 1. Values of percentage of decision-making for pre- and post-test in each player. DM = Decision-making; C1 = Control Player 1; C2 = Control Player 2; C3 = Control Player 3; C4 = Control Player 4; C5 = Control Player 5; C6 = Control Player 6; E1 = Experimental Player 1; E2 = Experimental Player 2; E3 = Experimental Player 3; E4 = Experimental Player 4; E5 = Experimental Player 5. Graphic 2. Values of total concepts in problem representation for pre- and post-test in each player. Tot Concept = Total of concepts; PR = Problem Representation; Abbreviations in X-axis are described in Graphic 1. Graphic 3. Values of total regulatory concepts in problem representation for pre- and post-test in each player. Tot Reg = Total regulatory concepts; PR = Problem Representation; Abbreviations in X-axis are described in Graphic 1. Graphic 4. Values of variety of regulatory concepts in problem representation for pre- and post-test in each player. Var Reg = Variety of regulatory concepts; PR = Problem Representation; Abbreviations in X-axis are described in Graphic 1. Graphic 5. Values of goal level 1 concepts in problem representation for pre- and post-test in each player. Goal-1 = Goal Level 1 concepts; PR = Problem Representation; Abbreviations in X-axis are described in Graphic 1. Graphic 6. Values of condition level 3 concepts in problem representation for pre- and post-test in each player. Cond-3 = Condition Level 3 concepts; PR = Problem Representation; Abbreviations in X-axis are described in Graphic 1. Graphic 7. Values of action level 3 concepts in problem representation for pre- and post-test in each player. Act-3 = Action Level 3 concepts; PR = Problem Representation; Abbreviations in X-axis are described in Graphic 1. Graphic 8. Values of number of linkages in problem representation for pre- and post-test in each player. PR = Problem Representation; Abbreviations in X-axis are described in Graphic 1. Graph [file pone.0082270.s002.doc]

**Appendix 1. Value graphics of variables with significant differences.**


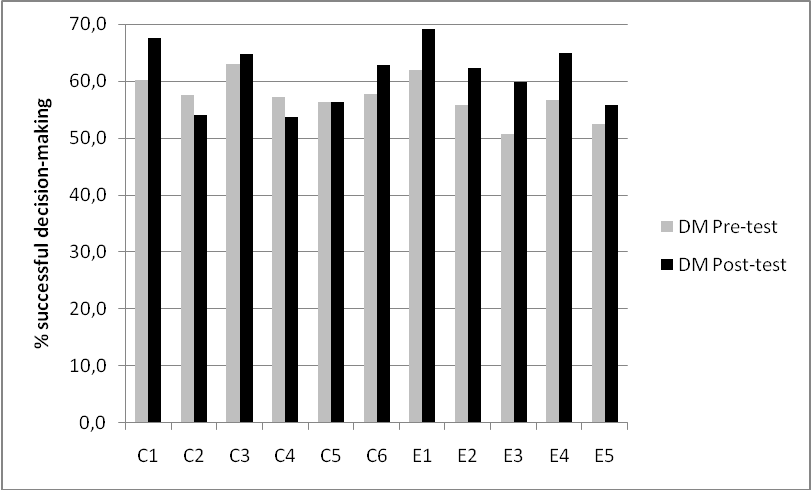


Graphic 1. Values of percentage of decision-making for pre- and post-test in each player.

DM = Decision-making; C1=Control Player 1; C2=Control Player 2; C3=Control Player 3; C4=Control Player 4; C5=Control Player 5; C6=Control Player 6; E1=Experimental Player 1; E2=Experimental Player 2; E3=Experimental Player 3; E4=Experimental Player 4; E5=Experimental Player 5.


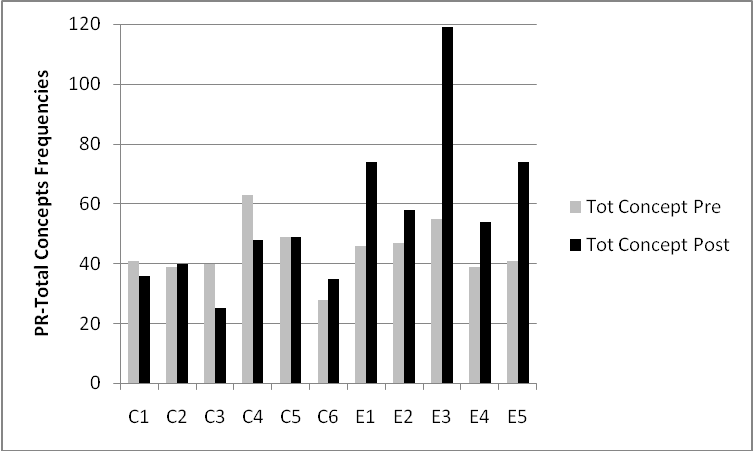


Graphic 2. Values of total concepts in problem representation for pre- and post-test in each player.

Tot Concept = total of concepts; PR=Problem Representation; Abbreviations in X-axis are described in Graphic 1.


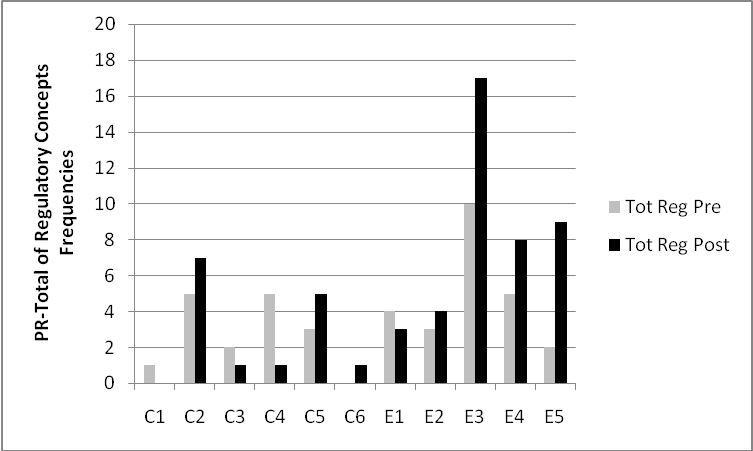


Graphic 3. Values of total regulatory concepts in problem representation for pre- and post-test in each player.

Tot Reg = Total regulatory concepts; PR=Problem Representation; Abbreviations in X-axis are described in Graphic 1.


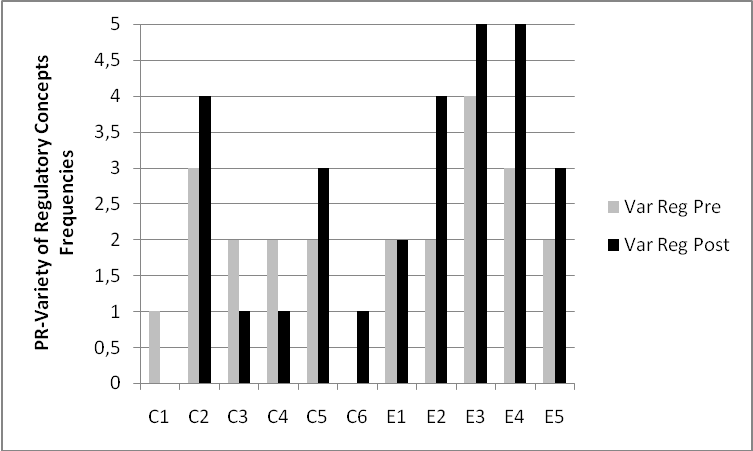


Graphic 4. Values of variety of regulatory concepts in problem representation for pre- and post-test in each player.

Var Reg=Variety of regulatory concepts; PR=Problem Representation; Abbreviations in X-axis are described in Graphic 1.


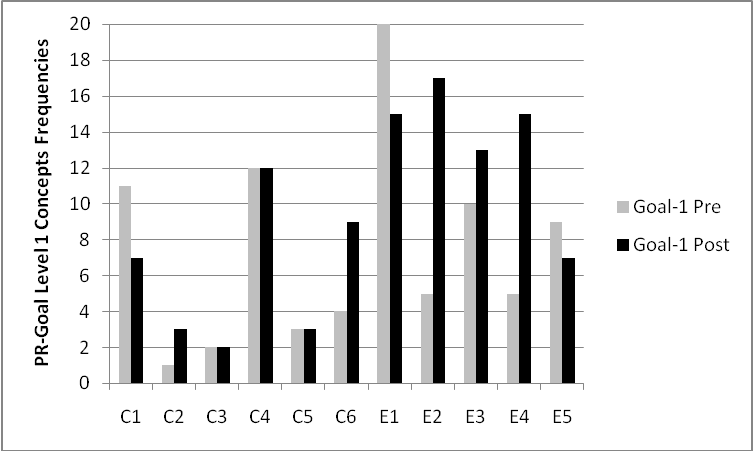


Graphic 5. Values of goal level 1 concepts in problem representation for pre- and post-test in each player.

Goal-1=Goal Level 1 concepts; PR=Problem Representation; Abbreviations in X-axis are described in Graphic 1.


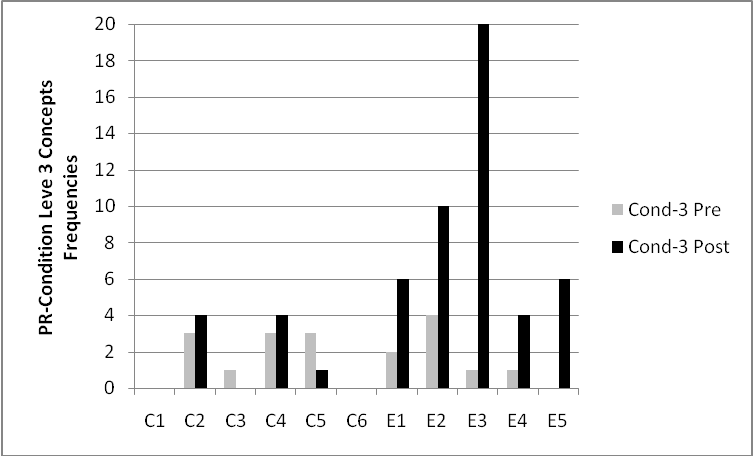


Graphic 6. Values of condition level 3 concepts in problem representation for pre- and post-test in each player.

Cond-3=Condition Level 3 concepts; PR=Problem Representation Abbreviations in X-axis are described in Graphic 1.


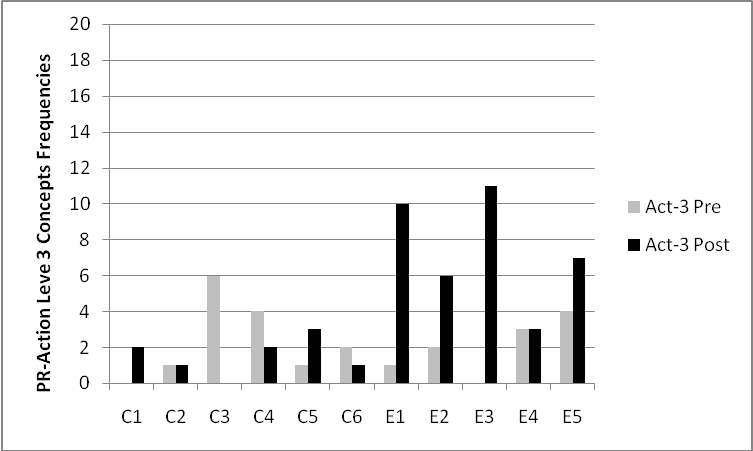


Graphic 7. Values of action-level 3 concepts in problem representation for pre- and post-test in each player.

Act-3=Action Level 3 concepts; PR=Problem Representation; Abbreviations in X-axis are described in Graphic 1.


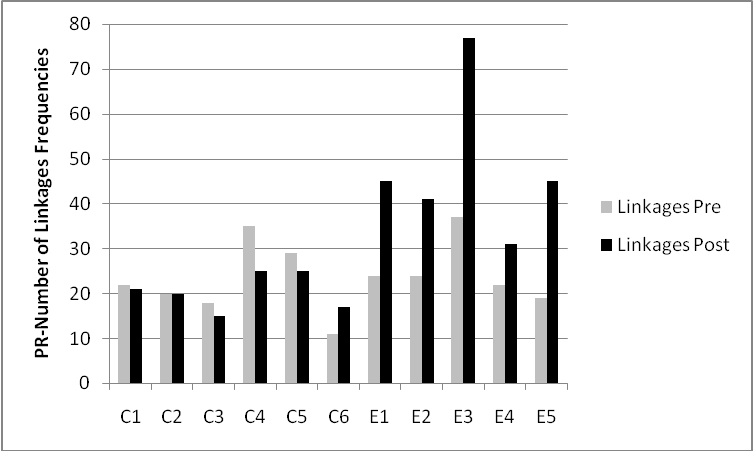


Graphic 8. Values of number of linkages in problem representation for pre- and post-test in each player.

PR=Problem Representation Abbreviations in X-axis are described in Graphic 1.


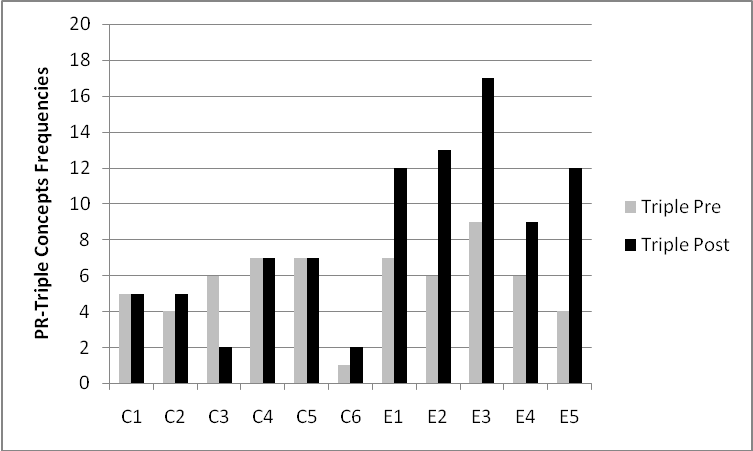


Graphic 9. Values of triple concepts in problem representation for pre- and post-test in each player.

Triple=Triple concepts; PR=Problem Representation; Abbreviations in X-axis are described in Graphic 1.


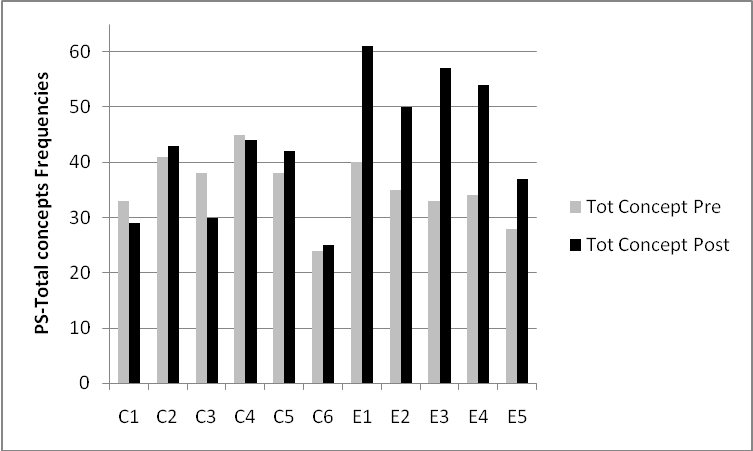


Graphic 10. Values of total concepts in planning strategy for pre- and post-test in each player.

Tot Concept=Total Concepts; PS=Planning strategy; Abbreviations in X-axis are described in Graphic 1.


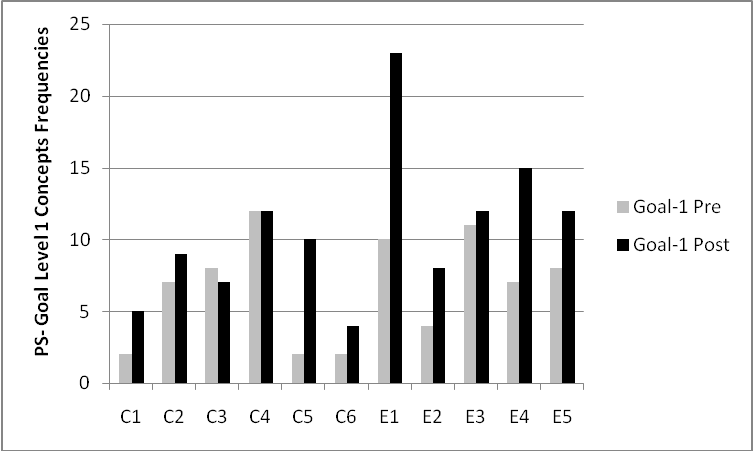


Graphic 11. Values of goal level 1 concepts in planning strategy for pre- and post-test in each player.

Goal-1=Goal Level 1; PS=Planning strategy; Abbreviations in X-axis are described in Graphic 1.


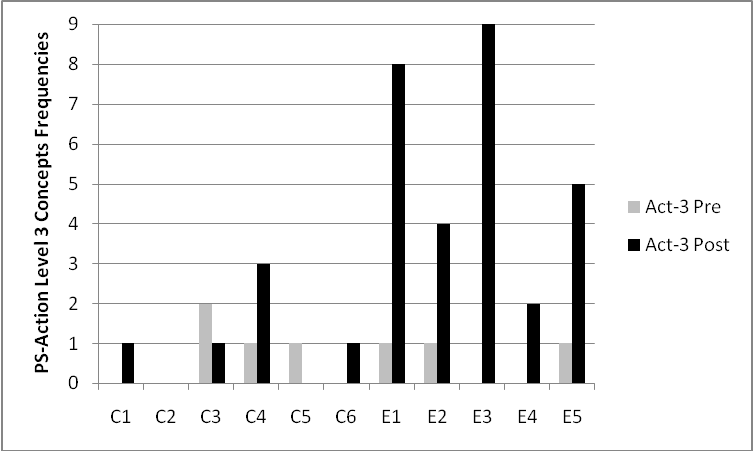


Graphic 12. Values of action level 3 concepts in planning strategy for pre- and post-test in each player.

Act-3=Action Level 3 concepts; PS=Planning strategy; Abbreviations in X-axis are described in Graphic 1.


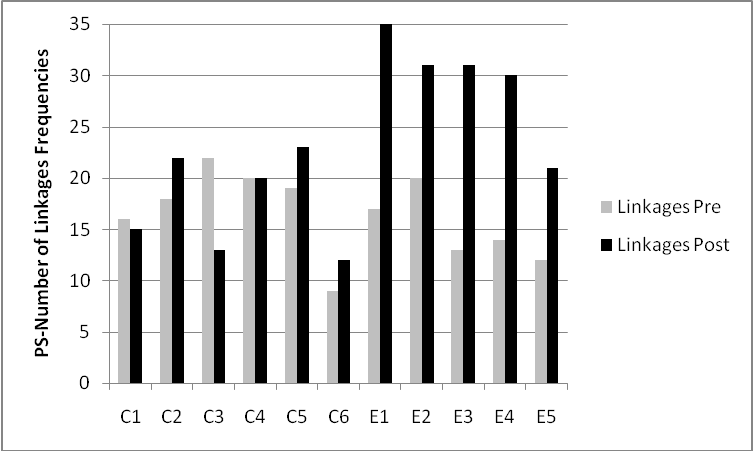


Graphic 13. Values of number of linkages in planning strategy for pre- and post-test in each player.

PS=Planning strategy; Abbreviations in X-axis are described in Graphic 1.


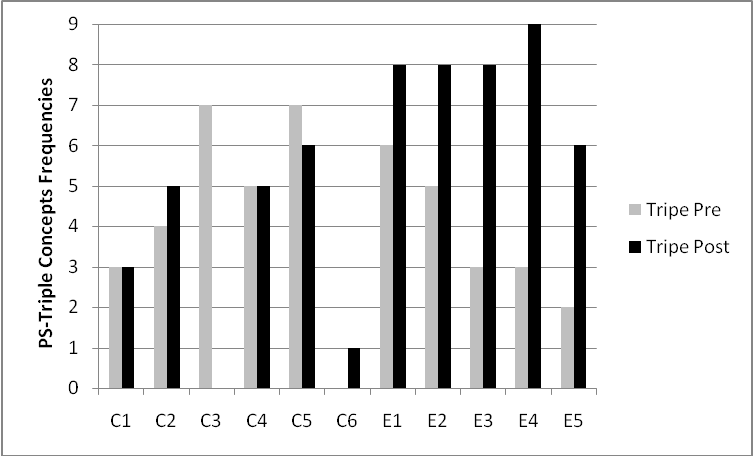


Graphic 14. Values of triple concepts in planning strategy for pre- and post-test in each player.

Triple=Triple concepts; PS=Planning strategy; Abbreviations in X-axis are described in Graphic 1.
